# Exosomes derived from hucMSC attenuate renal fibrosis through CK1δ/β-TRCP-mediated YAP degradation

**DOI:** 10.1038/s41419-020-2510-4

**Published:** 2020-05-07

**Authors:** Cheng Ji, Jiahui Zhang, Yuan Zhu, Hui Shi, Siqi Yin, Fengtian Sun, Qiongni Wang, Leilei Zhang, Yongmin Yan, Xu Zhang, Wenrong Xu, Hui Qian

**Affiliations:** 1 Zhenjiang Key Laboratory of High Technology Research on Exosomes Foundation and Transformation Application, Jiangsu Key Laboratory of Medical Science and Laboratory Medicine, School of Medicine, Jiangsu University, Zhenjiang, Jiangsu 212013 China; 2 Department of Clinical Laboratory Medicine, the Affiliated People’s Hospital of Jiangsu University, 212002 Zhenjiang, China

**Keywords:** Mechanisms of disease, Obstructive nephropathy

## Abstract

Exosomes from human umbilical cord mesenchymal stem cells (hucMSC-Ex) have been suggested as novel nanomaterials for regenerative medicine. Here we explored the roles of hucMSC-Ex through regulating Yes-associated protein (YAP) in renal injury repair by using rat unilateral ureteral obstruction (UUO) models. Our study identified mechanical stress induced YAP nucleus expression and stimulated collagen deposition and interstitial fibrosis in the kidney. Then, infusion with hucMSC-Ex promoted YAP nuclear cytoplasmic shuttling and ameliorated renal fibrosis in UUO model. Interestingly, hucMSC-Ex delivered casein kinase 1δ (CK1δ) and E3 ubiquitin ligase β-TRCP to boost YAP ubiquitination and degradation. Knockdown of CK1δ and β-TRCP in hucMSC decreased the repairing effects of hucMSC-Ex on renal fibrosis. Our results suggest that hucMSC-Ex attenuates renal fibrosis through CK1δ/β-TRCP inhibited YAP activity, unveiling a new mechanism for the therapeutic effects of hucMSC-Ex on tissue injury and offering a potential approach for renal fibrosis treatment.

## Introduction

The development of renal interstitial fibrosis (RIF) is linked to progressive renal injury and chronic kidney disease (CKD)^1^. And tubulointerstitial fibrosis is a very important and common pathological change in the progress of CKD, which seriously affects the prognosis of kidney disease^2^. Initiated by diabetes, obstruction, and hypertension, it is characterized by increased production of growth factors and inflammatory factors^3,4^. Its main pathological features are inflammatory cell infiltration, tubular atrophy, capillary loss and accelerated proliferation of myofibroblasts, and excessive deposition of extracellular matrix (ECM)^5,6^. In recent years, more and more evidences show that EMT changes of renal tubules are the main pathway of myofibroblasts production in renal diseases^7^. Long-term tubulointerstitial fibrosis reduced the regenerative potential of the kidneys and led to a substantial decline in renal function^8^. At present, there is lack of effective treatment that can prevent the renal fibrosis progression^9,10^. Thus, it is imperative to enhance the understanding towards the pathogenesis of tubulointerstitial fibrosis and consequently find new therapeutic approaches^11,12^.

Mesenchymal stem cells (MSCs) are multipotent adult stem cells that have been widely used in tissue regeneration^13,14^. MSCs have been reported to improve renal function, reduce renal damage, and inhibit chronic renal fibrosis^15–17^, where tissue damage was mainly repaired through paracrine mechanism^18^. Exosomes are cell-secreted membranous nano vesicles that mediate cell communication by delivering a variety of molecules, including nucleic acids, proteins and lipids, from donor cells to target cells^19,20^. MSC-derived exosomes have been shown to exert therapeutic effects on spinal cord injury^21^, multiple sclerosis^22^, infarcted hearts^23^. Our research focused on human umbilical cord MSC-derived exosomes (hucMSC-Ex), it was beneficial to liver fibrosis^24^, diabetic melitus^25^, and skin burn^26^. HucMSC-Ex could repair cisplatin-induced AKI by ameliorating oxidative stress and cell apoptosis, promoting cell proliferation in vivo and in vitro. hucMSC-Ex pretreatment promoted autophagy of renal tubular epithelial cells and reduced cisplatin-induced renal toxicity by transporting 14-3-3ζ protein^27,28^. However, whether hucMSC-Ex could attenuate renal fibrosis remains to be studied.

Being a key pathway involved in CKD progression, Hippo signaling controls organ size and regulates tissue regeneration^29,30^. Along the Hippo pathway yes-associated protein (YAP) is an important effector protein, playing an indispensable role in fibrosis^31^. As a co-factor YAP regulates TGF-β_1_ signaling by retaining activated Smad2/3 in the nucleus^32,33^. YAP expression could be upregulated by a variety of factors such as mechanical forces, leading to activation of myofibroblasts and excessive deposition of ECM^34^. YAP was a tissue mechanosensor that the interaction of YAP and ECM formed a feed-forward loop resulting in kidney fibrosis^35^, indicating that YAP may be a new target for anti-fibrosis therapy. CK1δ and β-TRCP were the kinase ubiquitin system of YAP protein degradation. Firstly, the protein substrate YAP was phosphorylated by CK1δ kinase at the serine site, and then recognized by E3 ubiquitin ligase transprted ubiquitin molecules which binded to the substrate, then hydrolyzed by proteasomes and promoted YAP degradation. These led us to research the intrinsic relationship between hucMSC-Ex, kinase ubiquitin system and YAP.

In this study we explored whether hucMSC-Ex could alleviate renal fibrosis induced by mechanical stress (unilateral ureteral obstruction (UUO) model). HucMSC-Ex infusion in rats showed that it could migrate to damaged kidney tissue and alleviated the damage of kidney tissue. Our results showed that hucMSC-Ex could transport CK1δ and β-TRCP system to promote YAP ubiquitination and degradation, therefore inhibiting YAP activation, lessening collagen deposition, and alleviating renal fibrosis. These findings provide a new strategy for therapeutic treatment of renal fibrosis.

## Material and methods

### UUO rat model

The SD rats (male) age were 8 weeks and the weight were 250 g. The temperature of the housing condition was kept at 25 °C, the relative humidity was 50%, which provided 12 h of light and 12 h of darkness. After 14 days, SD rats were anesthetized with 10% chloral hydrate. Briefly, under general anesthesia, the left ureter was ligated at the ureter-pelvic junction with 4-0 silk through a left flank incision. The right kidney was sham operated and the ureter was not ligated as a control. After intervention, all animals were euthanized and their kidneys were harvested on the 14th day after operation. Normal rats were fed with regular diets. Animal Ethics Committee of the University of Jiangsu approved all animal protocols (2014280).

### Isolation and characterization of exosomes

Exosomes were extracted and purified as previously described^23^. The protein concentration, as the quantification of exosomes, was determined by using a BCA protein assay kit (CWBIO, Beijing, China). The final amount of exosomes used for in vitro cell study was 160 μg/ml. For in vivo animal study, 200 μg exosomes were used for each animal. The morphology of the extracted exosomes was observed by using transmission electron microscopy (FEI Tecnai 12, Philips, Netherlands). The size of exosomes was analyzed by measuring the rate of Brownian motion using the NanoSight LM10 system (nanosight tracking analysis, UK).

### Cryo-TEM

A cryo-TEM observation of exosomes solutions was carried out in a controlled-environment vitrification system. The climate chamber temperature was 25–28 °C, and the relative humidity was kept close to saturation to prevent evaporation from the sample during preparation. Two milliliters exosomes sample solution at room temperature was placed on a carbon-coated holey film supported by a copper grid and gently blotted with filter paper about 3 s to obtain a thin liquid film (20–200 nm) on the grid. The grid was quenched rapidly in liquid ethane at −180 °C and then transferred to liquid nitrogen (−196 °C) for storage. The acceleration voltage was 200 kV, and the working temperature was kept below −170 °C. The images were recorded digitally with a charge-coupled device camera (Gatan) under low-dose conditions with an under focus of approximately 3 μm.

### Cell culture

Umbilical cords were obtained from the affiliated hospital of Jiangsu University with the permission of mothers and were freshly processed within 2 h. HucMSCs were isolated as previously described^24^, and human lung fibroblasts Cells (HFL1) maintained in a low-glucose Dulbecco’s modified Eagle medium (DMEM) containing 10% fetal bovine serum (Excell Bio, Australia). Rat renal proximal tubular (NRK-52E) cells were purchased from Stem Cell Bank of Chinese Academy of Sciences and maintained in H-DMEM (Gibco, USA) containing 10% fetal bovine serum and 1% penicillin and streptomycin at 37 °C with 5% CO_2_. The cells at passage 3 were used for the following studies.

### Preparation of polyacrylamide hydrogels

Hydrogels were prepared as previously described on 25 mm coverslips were utilized^36^. Briefly, firstly glass coverslips were etched using 0.1 NaOH, rinsed with ddH_2_O, incubated in 0.5% gluteraldehyde in phosphate-buffered saline (PBS), and then acrylamide/bis-acrylamide mixtures polymerized between the functionalized coverslip and a glass slide coated with dichlorodimethylsiloxane (Sigma-Aldrich).

### Histology and immunohistochemistry

For histologic analysis, the kidneys were prepared by perfusion of the rat through the left ventricle and slides of the kidney were prepared, fixed in 4% paraformaldehyde, embedded in paraffin and then cut into sections. The sections were stained with hematoxylin and eosin (HE) and Sirius Red and Masson staining, and the histological changes of renal tissues were observed under a microscope (DP73; Olympus, Tokyo, Japan). The expression and localization of YAP (0.6 μg/ml, CST, USA), α-SMA (0.5 μg/ml, CST, USA), CK1δ (1.0 μg/ml, CST, USA), and β-TRCP (1.0 μg/ml, CST, USA) in paraffin-embedded sections were measured as previously described^25^.

### Immunofluorescence staining

Renal tissue section placed in 4% paraformaldehyde at 4 °C for 12 h. Then permeabilized with PBS solution containing 0.15% Triton X-100 for 30 min and incubated with 5% bovine serum albumin (BSA) for 1 h to block non-specific antibody binding. Kidney slices were then incubated with the following primary antibodies: YAP (0.6 μg/ml, CST), α-SMA (0.5 μg/ml, CST), Collagen I (0.5 μg/ml, CST) at 4 °C for 24 h. The slices were washed with PBS and then incubated with two secondary antibody, Alexa Fluor 555-conjugated donkey anti-mouse IgG and FITC-conjugated goat anti-Rabbit IgG (0.1 μg/ml, Invitrogen, USA) overnight at 4 °C. The nucleus was stained with Hoechst33342 (1:200, Sigma, USA). The slides were visualized with a confocal microscope (DeltaVision Elite, GE, USA).

### Western blot analysis

Total protein was extracted from tissues and cells by using RIPA lysis buffer with proteinase inhibitors. The protein concentration was determined by using a BCA protein assay kit. Equal amounts of protein were separated on 12% SDS-PAGE gel and then transferred onto polyvinylidene fluoride (PVDF) membranes. After blockade with 5% skim milk for 1 h, the membranes were incubated with primary and the HRP-conjugated secondary antibodies and detected by using ECL detection system (Amersham Pharmacia Biotech, Little Chalfont, UK). The primary antibodies were as follows: CD9, CD63, Alix (50 ng/ml, CST, USA), α-SMA (100 ng/ml, BioWorld, USA), TGF-β_1_, Collagen I, FAP (100 ng/ml, SAB, USA), YAP, CK1δ (120 ng/ml, CSTc), β-TRCP (200 ng/ml, CST, USA), β-actin (50 ng/ml, CWBIO, China). The secondary antibodies were HRP-conjugated goat anti-rabbit and goat anti-mouse antibodies (10 ng/ml, CWBIO, China).

### Immunoprecipitation assays

Cells were pretreatment 5 h with 20 µM MG132 before immunoprecipitation. Cells were washed using PBS and subsequently lysed in Western/IP lysis buffer (Bioword, China), followed by addition of carrier beads. Immunocomplexes were washed five times with NETN buffer before being resolved by SDS-PAGE and immunoblotted with indicated antibodies.

### Adenovirus mediated knockdown of CK1δ and β-TRCP in hucMSCs

The adenovira CK1δ shRNA and β-TRCP shRNA vector were generated by the vector ADV1(U6/CMV-GFP) with CK1δ and β-TRCP shRNA oligonucleotides.

CK1δ shRNA oligonucleotide sequences are: 5′-GGGCAAGCTCTATAAGATTCTTCCTCGAGGAAGAATCTTATAGAGCTTGCTTTTT-3′. β-TRCP shRNA oligonucleotide sequences are: 5′-CCGGGCGTTGTATTCGATTTGATAACTCGAGTTATCAAATCGAATACAACGCTTT-3′. The sequences of control shRNA are: 5′-CCGGGCAAGCTGACCCTGAAGTTCATCTCGAGATGAACTTCAGGGTCACGTTGCTTTTTG-3′. Recombinant adenovirus was produced by co-transfecting HEK293T cells with ADV1-CK1δ-shRNA or ADV1-β-TRCP-shRNA and PU1563 plasmid using Lipofectamine2000 (Invitrogen, Shanghai, China). The efficiency of CK1δ an-d β-TRCP knockdown was evaluated by using real-time quantitative RT-PCR and western blot. HucMSCs were transduced with the prepared adenovirus (Adeno-CK1δ-shRNA, Adeno-β-TRCP-shRNA). The stable cell lines were cultured in serum-free medium for 48 h, the supernatants were collected and exosomes were isolated for further study.

### Statistical analysis

The data were analyzed using GraphPad Prism 5 (GraphPad, USA). All data are reported as the means ± standard deviation. Significant differences were evaluated using an unpaired Student’s *t*-test for the comparison of two groups and one-way ANOVA Tukey test for multiple-group comparisons. *P < 0.05 was considered significant.

## Results

### HucMSC-Ex alleviated renal fibrosis in UUO rats model

**Fig. 1:**
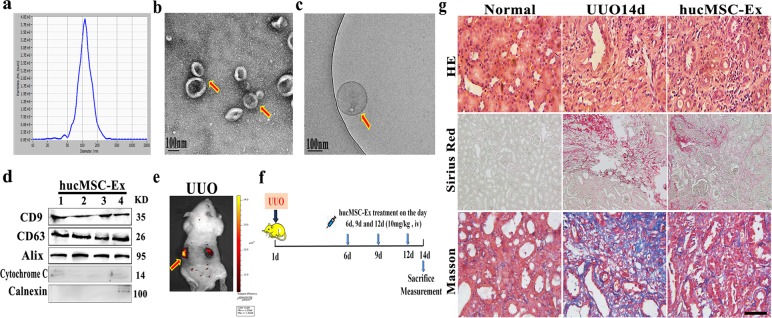
HucMSC-Ex treatment reduced renal interstitial fibrosis. **a** The size of hucMSC-Ex was measured by NTA. **b** The morphology of hucMSC-Ex was identified by TEM. **c** The images of hucMSC-Ex were detected by cryo-EM. **d** Western blot assay for exosomal markers CD9, CD63, and Alix in hucMSC-Ex. **e** CM-DiR labeled hucMSC-Ex (10 mg/kg) were intravenously administrated into 14d UUO rats. The distribution of CM-DiR labeled hucMSC-Ex in the kidney detected by IVIS imaging system. **f** Schematic of UUO induction and exosomes treatment. UUO rats were intravenously injected with exosomes (10 mg/kg/injection) on days 6, 9, and 12. The rats were sacrificed on day 14 for subsequent experiments (*n* = 10). **g** Representative images of HE staining, Sirius red and Masson staining of kidneys in 14d UUO rats treated with hucMSC-Ex. Bar = 100 μm.

HucMSC-Ex were first extracted and isolated as previously described^23^. As nanoscale membrane vesicles, hucMSC-Ex size was determined to be 106.3 ± 37.3 nm (mean ± SD) by nanoparticle tracking analysis (NTA) (Fig. 1a). Exosomes were further characterized by using transmission electron microscopy (TEM) and displayed cup-like spherical vesicles (point by red arrow) (Fig. 1b), cryo-TEM (cryo-TEM) graphic of exosomes were small circular structures vesicles (Fig. 1c). Western blot results confirmed the expression of exosomal marker CD9, CD63, and Alix in hucMSC-Ex, and negative for cytochrome *C*, calnexin (Fig. 1d). To investigate the roles of hucMSC-Ex in kidney fibrosis, firstly, the bio-distribution of hucMSC-Ex in vivo was detected via IVIS Lumina system. CM-DiR-labeled hucMSC-Ex were injected into UUO rats via tail vein, and observed the fluorescent signal in the damaged kidney tissues at 24 h post injection (Fig. 1e). Interestingly, the kidney tissue ligated on the left side, and the fluorescence intensity was 15 times higher than the control side (Fig. 1e). These information suggesting that hucMSC-Ex as nanometer vesicles could efficiently enter the damaged kidney.

In order to confirm the therapeutic components of anti-fibrosis, with hucMSC-Ex treatment in UUO rat model (Fig. 1f), though infused hucMSC-CM, hucMSC-Ex, and human lung fibroblast-ex (HFL1-Ex) into the injured rats, separately. The results of immunohistochemistry (α-SMA) and immunofluorescence (collagen I) of renal tissue confirmed that hucMSC-Ex was the effective ingredients in the repair of renal fibrosis (Supplementary Fig. S1a). The results of HE and Sirius red staining showed that the glomerular basement membrane of kidneys in 14d UUO rats was significantly thickened compared those in normal rats. Masson staining results revealed a large amount of collagen fibers in the renal interstitium of 14 days UUO rats. Following hucMSC-Ex treatment, the extent of interstitial fibrosis was significantly lessened (Fig. 1g). In the kidney tissues of UUO, fibrosis-related proteins (Collagen I, FAP, α-SMA, TGF-β_1_) were significantly reduced after hucMSC-Ex intervention (Fig. S1b). The serum urea nitrogen levels and the urinary albumin to creatinine ratio were increased as the disease progressed, but hucMSC-Ex treatment could maintain the renal function at a certain level (Fig. S1c, d). These results indicated that hucMSC-Ex could ameliorate renal fibrosis, delaying disease progression.

### Mechanical stiffness activated YAP in the kidneys of UUO rats

**Fig. 2:**
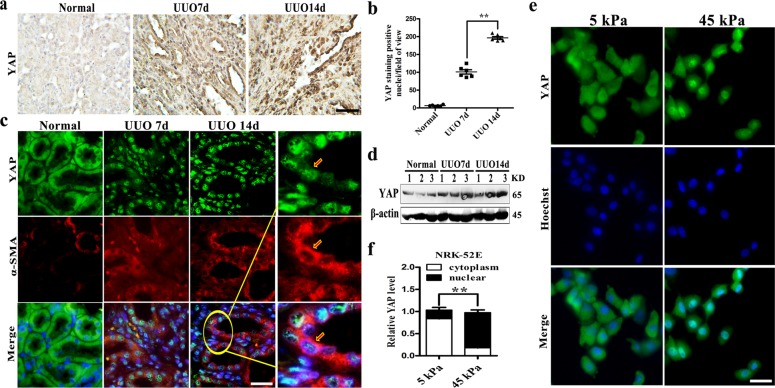
Sustained mechanical stress induced YAP activation and aggravated renal fibrosis. **a** YAP immunohistochemistry staining of UUO 7d, 14d kidney sections. Bar = 100 μm. **b** Quantification of YAP-positive nuclei per field of view from the **a** experiment. **c** Double immunofluorescent staining of YAP and α-SMA in the kidneys of control and UUO (at 7 and 14 days) rats (Green: YAP; Red: α-SMA). Bar = 50 μm. **d** Western blot assay for YAP protein expression in the kidneys of control and UUO rats (at 7 and 14 days) rats (*n* = 3). **e** Nuclear localization of YAP in NRK-52E cells treated with different stiff gel for 48 h was detected by confocal microscopy. Bar=25 μm. **f** Statistical analysis the expression of YAP in cytoplasm and nuclear under mechanical pressure. **P < 0.01.

YAP is a key transcription co-factor in Hippo pathway and its dysfunction is involved in the pathogenesis of various diseases^37,38^. However, YAP protein as a fibrogenesis promoter, and the exact role of YAP in renal fibrosis remains not clear. Firstly, in UUO rat kidney, immunohistochemistry staining showed YAP nucleus expression (Fig. 2a), and then as the disease progressed, YAP increased dramatically (Fig. 2b). Continuous mechanical stress stimulation YAP was found to be accumulated in the nucleus of tubular epithelial cells and co-localized with α-SMA, an indicator of renal fibrosis, being associated with renal fibrosis progression (Fig. 2c). Western blot showed that the up-regulation and activation of YAP were also observed at 7 and 14 days after injury (Fig. 2d). In vitro model, we employed the 2D polyacrylamide (PA) hydrogel system with calibrated elastic moduli ranging from the soft gel (5 kPa) of normal kidney to the stiff gel (45 kPa) of fibrosis renal^39^. Simultaneously, increased expression and nuclear distribution of YAP were also found in cultured rat renal tubular epithelial cells (NRK-52E) after being stimulated with stiff gel (45 kPa) for 48 h (Fig. 2e), and statistical analysis showed that YAP entered the nucleus about 87% under 45 kPa stiff gel (Fig. 2f). Those results indicated that YAP could be activated by mechanical stress in UUO rat models. YAP enters the nucleus, facilitates the expression of α-SMA and induced renal fibrosis, which may be involved in the progression of CKD.

### HucMSC-Ex inhibited YAP expression in UUO rat models

**Fig. 3:**
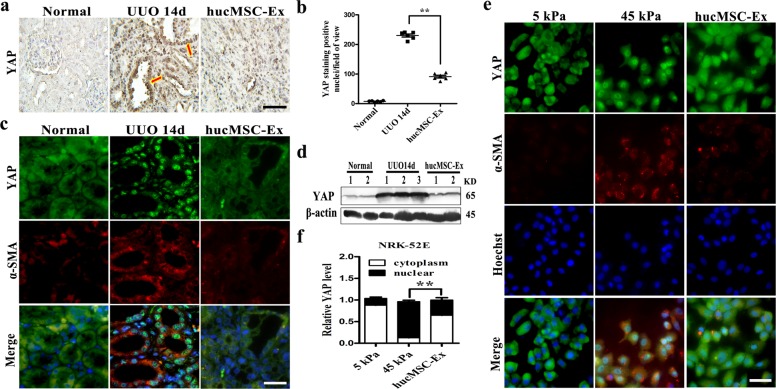
HucMSC-Ex attenuated tubulointerstitial fibrosis by inhibited YAP. **a** YAP immunohistochemistry staining of 14d UUO kidney with hucMSC-Ex treatment. Bar=100 μm. **b** Quantification of YAP-positive nuclei per field of view from the **a** experiment. **c** Double immunofluorescent staining of YAP and α-SMA in the kidney of 14d UUO rats treated with hucMSC-Ex (Green: YAP; red: α-SMA). Bar = 50 μm. **d** Western blot analyses of YAP expression in the kidney of 14d UUO rats with hucMSC-Ex intervene (*n* = 3). **e** The co-localization of YAP and α-SMA in NRK-52E cells cultured under stiff gel condition in the presence of hucMSC-Ex was detected by double immunofluorescent staining. Bar=25 μm. **f** Statistical analysis the expression of YAP in cytoplasm and nuclear with hucMSC-Ex therapy. **P < 0.01.

Considering the critical role of YAP in the development of kidney diseases^29^, in this study, we found mechanical pressure stimulation induced YAP nuclear translocation, interact with Smad2/3 to induce α-SMA expression and aggravated fibrosis; however, we hypothesized that this negative regulation could be neutralized by hucMSC-Ex treatment by regulating YAP. The immunohistochemistry experiment revealed that decreased numbers of YAP in obstructed renal with hucMSC-Ex treatment (Fig. 3a), whereas statistical analysis significantly reduced the number of positive cells by 55% (Fig. 3b). The results of double immunofluorescent staining showed that YAP expression in the kidneys of 14d UUO model rats was inhibited by hucMSC-Ex treatment, which was accompanied with the decreased expression of α-SMA (Fig. 3c). Similarly, the renal lysate protein expression of YAP and α-SMA was decreased in 14d UUO model that had been treated with hucMSC-Ex by western blotting (Fig. 3d). In addition, hucMSC-Ex treatment also diminished the expression of YAP and fibrosis-related markers (α-SMA) in cultured NRK-52E cells under stiff gel condition (Fig. 3e), and statistical analysis showed that hucMSC-Ex significantly inhibited YAP protein from entering the nucleus (Fig. 3f). Hence, hucMSC-Ex might attenuate renal fibrosis by inactivating YAP to the extent.

### HucMSC-Ex transported CK1δ and β-TRCP to promoted YAP degradation

**Fig. 4:**
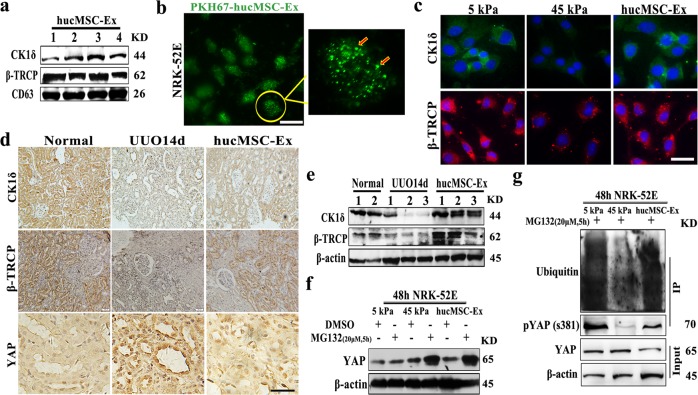
HucMSC-Ex delivered CK1δ and β-TRCP to promote YAP ubiquitination and degradation. **a** Western blot assay for CK1δ and β-TRCP proteins in hucMSC-Ex (*n* = 4). **b** The internalization of hucMSC-Ex (PKH67, green) in NRK-52E cells was observed by confocal microscopy. Bar = 25 μm. **c** The expression of CK1δ and β-TRCP in NRK-52E cells under mechanical stress. Bar = 25 μm. **d** The expression of CK1δ, β-TRCP, and YAP in the kidneys of 14d UUO rats with hucMSC-Ex treatment was determined by immunohistochemistry. Bar = 100 μm. **e** Western blot assay for CK1δ and β-TRCP proteins in the kidneys of 14d UUO rats treated with hucMSC-Ex (*n* = 3). **f** NRK-52E cells were pre-treated with MG132 (20 μm) for 5 h followed by stiff gel stimulation in the presence of hucMSC-Ex. YAP protein level was detected by western blot. **g** The ubiquitination of YAP protein with hucMSC-Ex treatment determined by co-IP.

YAP pathway was regulated by ubiquitination, the kinase ubiquitin system (CK1δ/β-TRCP) mediated YAP degradation^40^. To elucidate the mechanism by which hucMSC-Ex inhibited YAP expression and alleviated renal fibrosis, the protein profile of hucMSC-Ex were analyzed by LC-MS/MS, and then found the proteins related to ubiquitination/degradation proteome system were enriched in hucMSC-Ex (Supplementary Fig. S2a). In particular, we verified the expression of CK1δ and β-TRCP in hucMSC-Ex (Fig. 4a). In order to confirm that exosomes could be uptake by cells, with 24 h co-incubation, hucMSC-Ex which labeled with PKH67 (green) could be internalized into NRK-52E cells were measured by confocal microscopy (Fig. 4b). In vitro, matrix hardness induced the inactivation of kinase ubiquitin system, and hucMSC-Ex intervention restored its level (Fig. 4c). Thereafter, the expression of CK1δ and β-TRCP was declined in the UUO kidneys but elevated after hucMSC-Ex treatment (Fig. 4d), and the results of tissue protein were consistent with the results (Fig. 4e). When CK1δ/β-TRCP was inactivated, then the YAP expression was increased in the renal tubule and the decreased expression of YAP after hucMSC-Ex treatment. The similar changes in CK1δ and β-TRCP mRNA expression were also observed in vivo model after hucMSC-Ex treatment (Supplementary Fig. S2b–e). It was known that YAP phosphorylation was critical for its stability, and CK1δ/β-TRCP mediated YAP degradation^39^. We need to demonstrate that hucMSC-Ex regulated YAP stability via CK1δ and β-TRCP. Therefore, NRK-52E cells were pre-treated with proteasome inhibitor MG132 (20 μM), and then stimulated with stiff gel in the presence or absence of hucMSC-Ex, the down-regulation of YAP by hucMSC-Ex was restored by addition of MG132 (Fig. 4f). This experiment suggests that hucMSC-Ex might affect the stability of YAP protein, and the results of co-immunoprecipitation (co-IP) showed that hucMSC-Ex treatment significantly increased the ubiquitinated modification of YAP (Fig. 4g). Therefore, these data confirmed that hucMSC-Ex could delivery active molecules (CK1δ/β-TRCP) to target cells and participate in ubiquitination and degradation of YAP.

### CK1δ and β-TRCP knockdown impaired the therapeutic effect of hucMSC-Ex

**Fig. 5:**
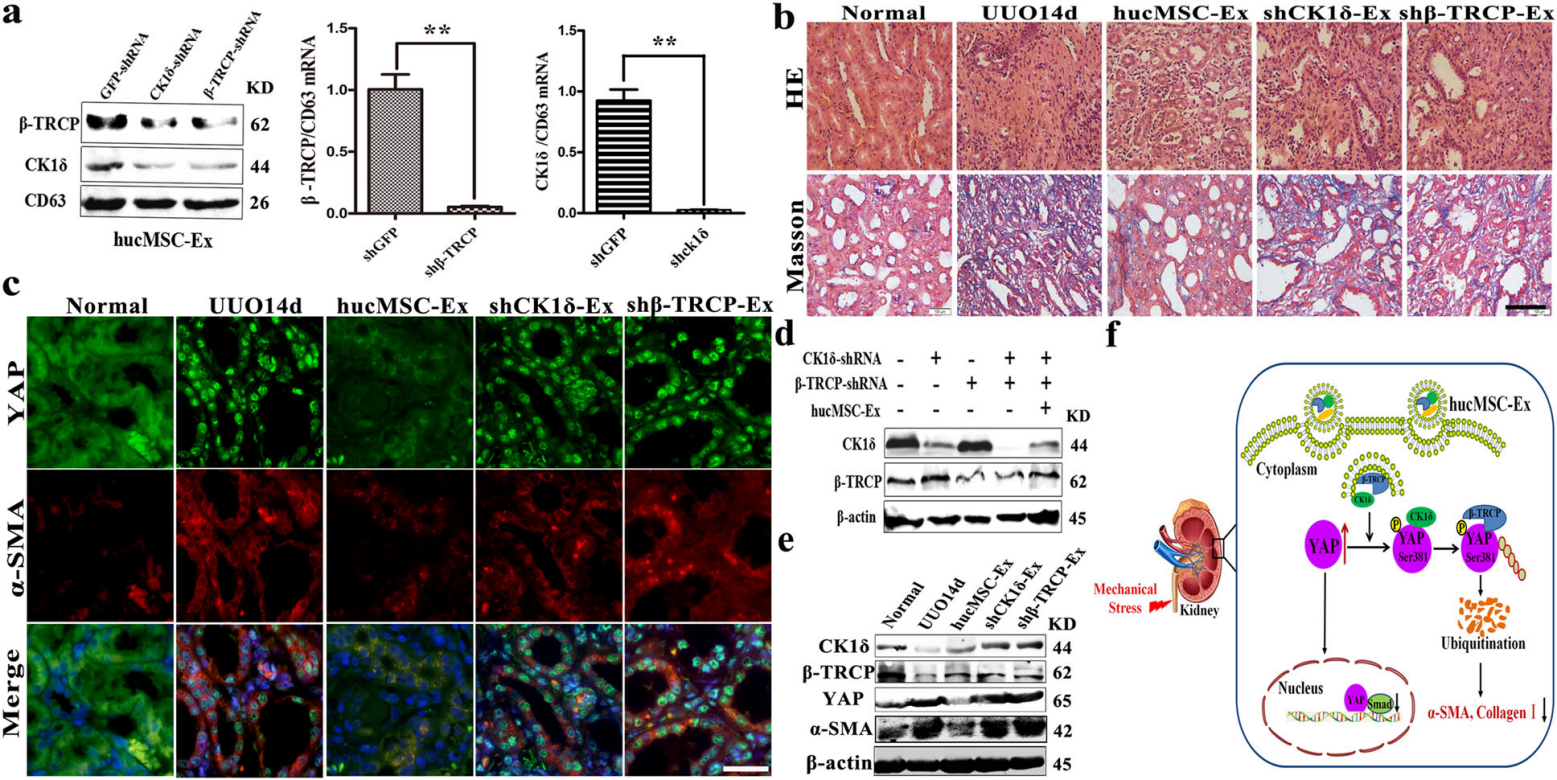
CK1δ and β-TRCP knockdown decreased anti-fibrosis effective of hucMSC-Ex. **a** Expression of CK1δ and β-TRCP in hucMSC-Ex was determined by western blot and qRT-PCR. **b** Representative images of HE and Masson staining in the UUO model with hucMSC-Ex, shCK1δ-Ex, and shβ-TRCP-Ex. Bar = 100 μm. **c** Double immunofluorescent staining of YAP and α-SMA in the kidneys of the UUO model treated with hucMSC-Ex, shCK1δ-Ex, and shβ-TRCP-Ex. Bar = 50 μm. **d** Western blot analyses for CK1δ and β-TRCP in NRK-52E after hucMSC-Ex treatment. **e** The expression of CK1δ, β-TRCP, YAP, and α-SMA in UUO14d with hucMSC-Ex, shCK1δ-Ex, and shβ-TRCP-Ex treatment was detected by western blot. **f** A proposed hypothesis for hucMSC-Ex attenuated tubulointerstitial fibrosis through CK1δ and β-TRCP mediated YAP ubiquitination and degradation.

To evaluate the importance of CK1δ and β-TRCP in the repairing effect of hucMSC-Ex on renal fibrosis, we knocked down CK1δ and β-TRCP expression in hucMSC by using adenovirus-mediated shRNA transfection. Exosomes were isolated from control and specific shRNA-transfected hucMSC (shCK1δ-Ex and shβ-TRCP-Ex) (Fig. 5a), and then treatment in UUO rat models. HE staining and Masson staining results showed that compared with hucMSC-Ex group, the thickness of glomerular basement membrane and the deposition of collagen fibers in shCK1δ-Ex and shβ-TRCP-Ex groups increased significantly (Fig. 5b). More interestingly, the expression and nuclear localization of YAP and α-SMA were significantly increased in the kidneys of rats treated with shCK1δ-Ex and shβ-TRCP-Ex when compared to hucMSC-Ex (Fig. 5c). In parallel, we found that the treatment with shCK1δ-Ex and shβ-TRCP-Ex could not increase CK1δ or β-TRCP expression in NRK-52E cells as that observed for hucMSC-Ex (Fig. 5d). In the mechanical stress vivo environment, compared to hucMSC-Ex group, the expressions of YAP, α-SMA were increased, shCK1δ-Ex and shβ-TRCP-Ex had impaired ability to inhibit YAP expression in UUO rat model (Fig. 5e). Moreover, we have carried out the three different doses of hucMSC-Ex to confirm the therapeutic application potential (Supplementary Fig. S3d, e). These results suggested that the inhibitory role of hucMSC-Ex in YAP expression and the ensuing therapeutic effect on renal fibrosis were significantly impaired by CK1δ and β-TRCP knockdown. In summary, this study we found that hucMSC-Ex attenuated renal fibrosis by regulating YAP degradation via CK1δ and β-TRCP (Fig. 5f).

## Discussion

Renal fibrosis is caused by excessive accumulation of ECM, which is present in almost all types of CKD^41^. However, there are few effective treatments to prevent renal fibrosis. MSCs have been suggested as important seed cells for anti-fibrosis therapy in regenerative medicine^42,43^. Systemic injection of MSC has been shown to be effective in experimental tissue injury model studies^44^. MSCs transplantation have been used in clinical trials as a potential treatment for nephropathy^16^. The mechanisms for the therapeutic effect of MSCs are complicated and the secretion of active molecules has been suggested as a major reason^18^. Both MSC-derived microvesicles and exosomes have been suggested to protect against renal injury^18,45,46^. MSC-derived exosomes have been shown to repair tissue damage by transferring active molecules into target cells^47^, including mRNAs, microRNAs, and proteins. In this study, we demonstrated that hucMSC-Ex could increase CK1δ and β-TRCP expression and promote YAP ubiquitination and degradation in the kidneys of UUO rat models, which inhibited ECM deposition and alleviated renal fibrosis, indicating that hucMSC-Ex may represent a new strategy for the treatment of renal fibrosis.

Activation of YAP in the mouse can promote regeneration in poor capacity organs, remarkably, prolonged activation of YAP/TAZ triggers cancer development^48^. In this study we first constructed rodent disease models (UUO) to examine the alteration of YAP expression during renal fibrosis and consequently the therapeutic effect of hucMSC-Ex. In vivo and in vitro both studies showed that mechanical stress, high glucose, and TGF-β_1_ stimulation induced the expression of YAP in the kidneys. It is in agreement with previous research found that YAP expression and phosphorylation increased in proximal tubular epithelial cells in animal models of type 1 and type 2 diabetes^49^. As YAP activation increased the stiffness of ECM, which further stimulated the activation of YAP in turn, forming a feed-forward loop to promote scar tissue formation and renal fibrosis^35^. Here we showed that the expression of YAP increased with the aggravation of renal fibrosis in UUO models, indicating that YAP may be a potential target for anti-fibrosis therapy.

YAP expression and activity are governed by two mechanisms: cytoplasmic retention, ubiquitination, and degradation^40,50^. In mammalian cells, YAP phosphorylated by Lats1/2 kinase at Ser127 site could bind to 14-3-3 and anchor to the cytoplasm^51^. Our previously studies showed that 14-3-3ζ protein in hucMSC-Ex bound Ser127-YAP to retard cell proliferation and inhibit skin scar formation^52^. Ubiquitin proteasome was the most important protein degradation pathway. Ubiquitin ligase E3 which determined the specific recognition of the target protein, promoted the ubiquitin degradation of proteins, and played an important role in many physiological processes in cells. Once phosphorylated by CK1δ kinase at Ser381 site, YAP could interact with the E3 ubiquitin ligase β-TRCP, leading to its poly-ubiquitination and degradation^39^. In UUO rat models, hucMSC-Ex treatment reduced the expression of YAP, suggesting the regulation of YAP stability by hucMSC-Ex. Due to the fact that YAP protein stability is regulated by the ubiquitin–proteasome system, we analyzed the protein component of hucMSC-Ex by LC-MS/MS. It was found that CK1δ and β-TRCP, two major ubiquitination related enzyme for YAP degradation, were enriched in hucMSC-Ex. Systemically administrated hucMSC-Ex could target the damaged kidneys in UUO rats and increase the expression of CK1δ and β-TRCP. When CK1δ and β-TRCP were knocked down, the therapeutic effect of hucMSC-Ex on renal fibrosis was greatly reduced, which suggested that hucMSC-Ex mediated CK1δ and β-TRCP in the repair of renal injury was very important.

In summary we demonstrated that mechanical stress stimulated YAP expression and accelerated the progression of renal fibrosis. HucMSC-Ex could inhibit renal fibrosis by delivering CK1δ and β-TRCP to promote the ubiquitination and degradation of YAP. As a biogenic nanotherapeutics, hucMSC-Ex could be employed to the target tissues to inhibit YAP activity and ameliorate renal fibrosis, providing a novel and effective approach for anti-fibrosis therapy.

## Experimental section

Details of experimental methods and procedures are available in the Supporting Information.

## Supplementary information

Supplementary Figure legends

Figure S1

Figure S2

Figure S3
