# Supplementary material for: Exosomes derived from hucMSC attenuate renal fibrosis through CK1δ/β-TRCP-mediated YAP degradation
Source: Cell Death Dis. 2020 May 7;11(5):327. doi: 10.1038/s41419-020-2510-4 (PMC7205986; doi:10.1038/s41419-020-2510-4)
Supplement: Supplementary file 1 — Supplementary Figure legends [file 41419_2020_2510_MOESM1_ESM.docx]

**Supplementary Figure legends**

**Figure S1. HucMSC-Ex ameliorated renal interstitial fibrosis in rat model.**

**a)** Immunohistochemistry and immunofluorescent staining analyses of α-SMA, collagen I in 14d UUO kidney tissue with hucMSC-CM, hucMSC-Ex and HFL1-Ex treatment. Bar=100μm. **b**) Western blot analyses of fibrosis markers (collagen I, FAP, α-SMA, and TGF-β1) in UUO14d with hucMSC-Ex treatment. **c)** Serum urea nitrogen level in UUO rats. **d)** Changes in the albumin to creatinine ratio in urine (U-alb/Cr) after administration of hucMSC-Ex.

**Figure S2. HucMSC-Ex increased CK1δ and β-TRCP in CKD kidney tissue. a)** LC-MS/MS analysis results of hucMSC-Ex protein content. **b)** qRT-PCR analyses of the relative mRNA levels of CK1δ with hucMSC-Ex treatment. **c)** qRT-PCR analyses β-TRCP mRNA with hucMSC-Ex treatment. **d)** Immunofluorescent staining showed the expression of CK1δ **d)** and β-TRCP **e)** in the renal tissues of 14d UUO rats with hucMSC-Ex intervene. Bar=100μm.

**Figure S3. The dose of hucMSC-Ex reduced renal fibrosis effective**.

**a)** Quantification kidney tissues of YAP expression in 7d and14d in UUO rats. n=3 **b**) Quantification kidney tissues of YAP expression with hucMSC-Ex intervene. n=3 **c)** Quantification kidney tissues of CK1δ and β-TRCP expression with hucMSC-Ex treatment. n=3 **d)** Representative images of HE staining, Sirius red staining of kidneys in 14d UUO rats treated with different doses of hucMSC-Ex. n=6, Bar=100μm. **e)** Western blot analyses of fibrosis protein (collagen I, α-SMA, YAP, CK1δ and β-TRCP) in UUO14d with different doses of hucMSC-Ex treatment. **f)** Microscopical observation of single cell suspension in renal tissue. Bar=100μm. **g)** Immunofluorescent staining showed the co-localization of Slc5a 12 (red) and YAP (green) in the renal tubule cells of 14d UUO rats with hucMSC-Ex intervene. Bar=100μm.
