# Supplementary figures and images for: Exosomes derived from hucMSC attenuate renal fibrosis through CK1δ/β-TRCP-mediated YAP degradation
Source: Cell Death Dis. 2020 May 7;11(5):327. doi: 10.1038/s41419-020-2510-4 (PMC7205986; doi:10.1038/s41419-020-2510-4)

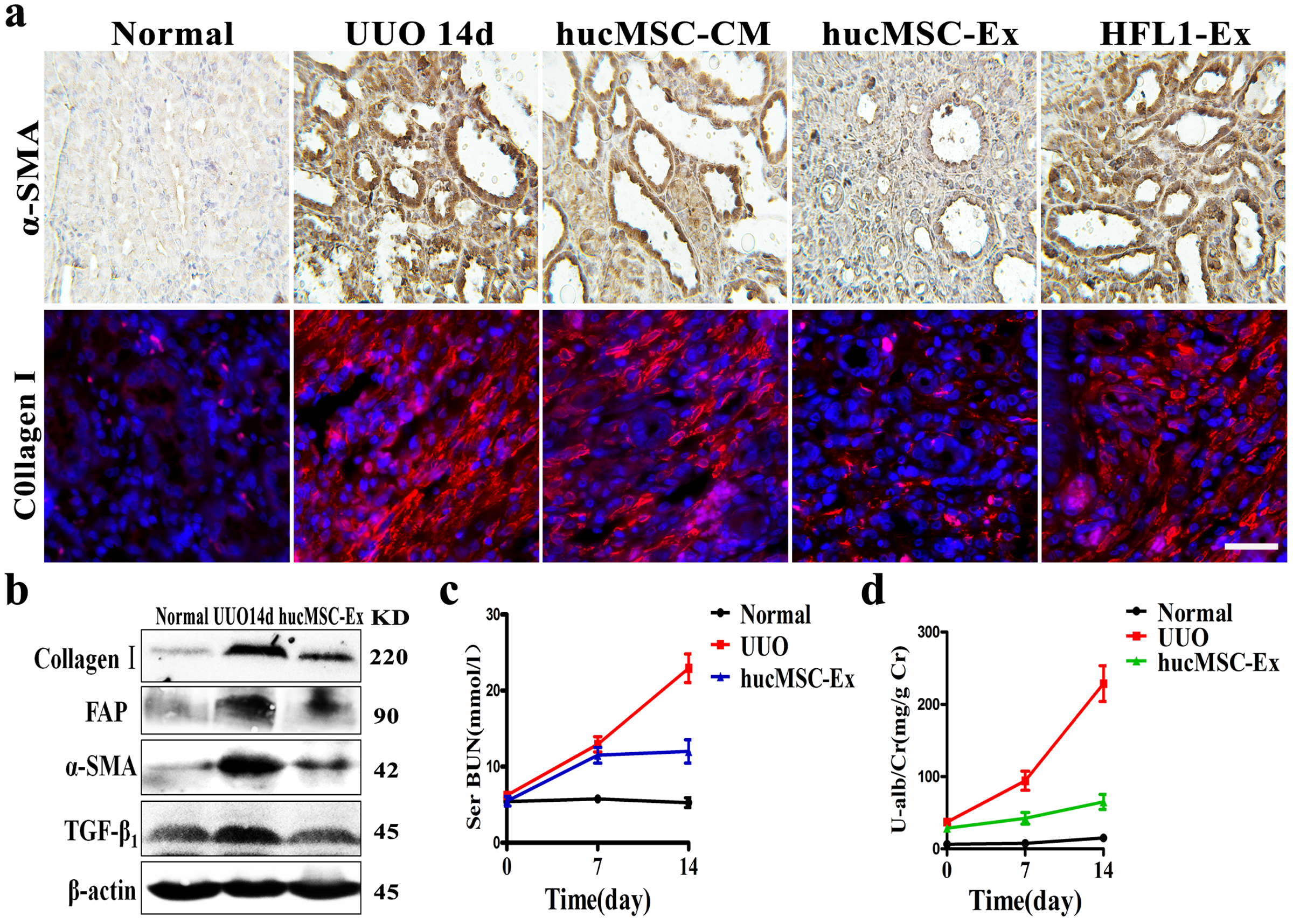

Supplement: Supplementary file 2 — Figure S1 [file 41419_2020_2510_MOESM2_ESM.tif]

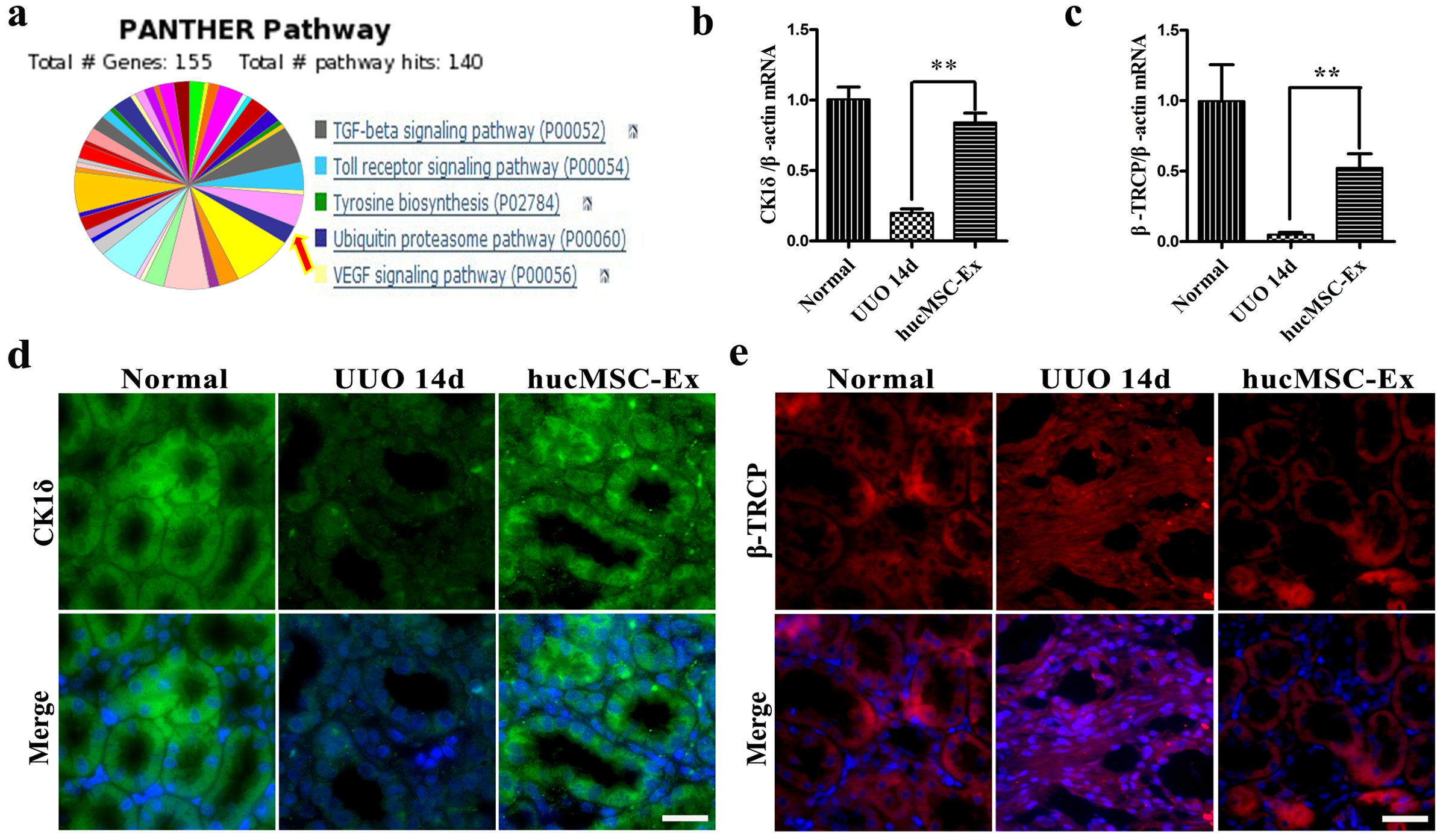

Supplement: Supplementary file 3 — Figure S2 [file 41419_2020_2510_MOESM3_ESM.tif]

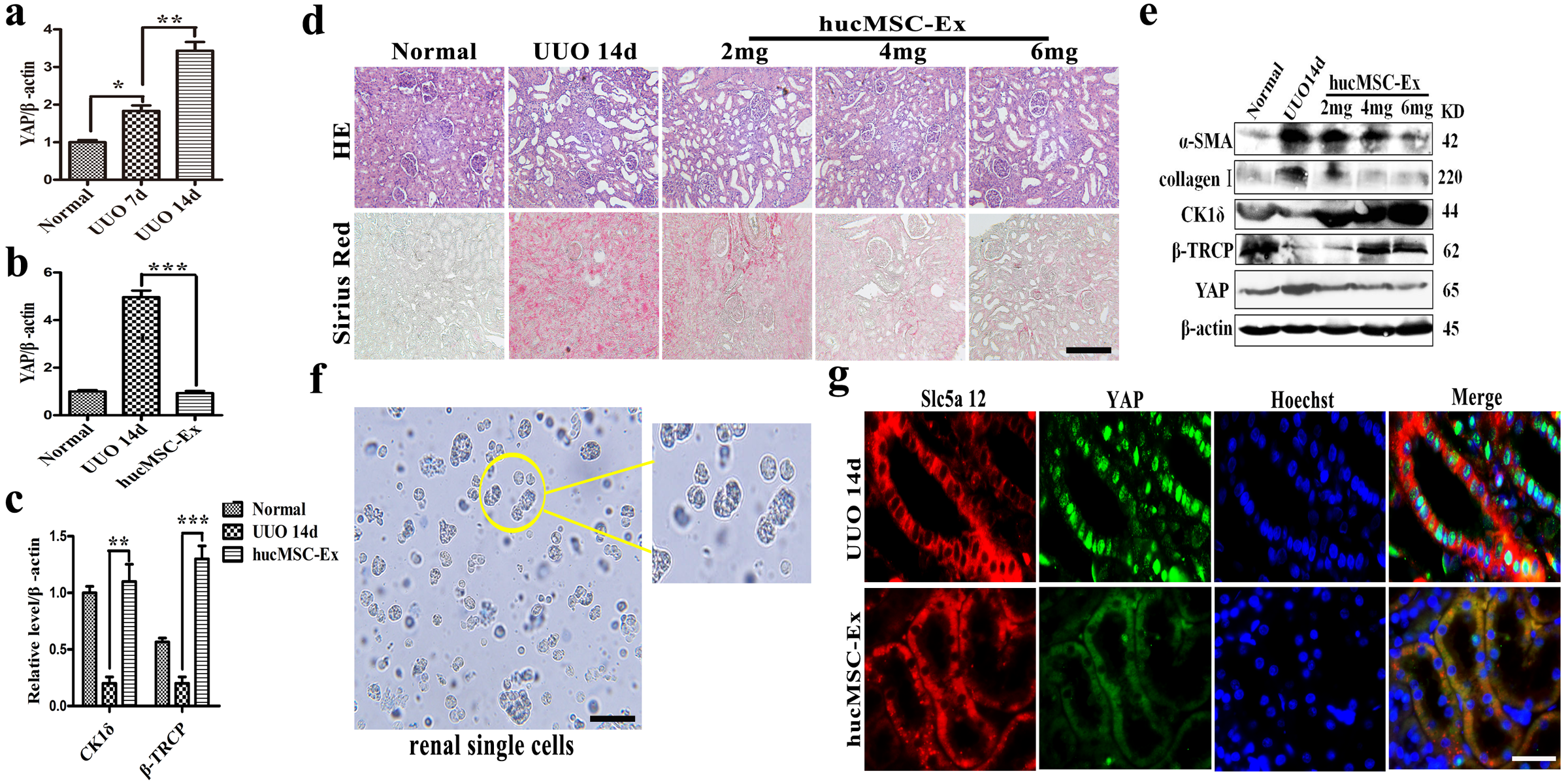

Supplement: Supplementary file 4 — Figure S3 [file 41419_2020_2510_MOESM4_ESM.tif]
